# Superimposed Pristine Limestone Aquifers with Marked Hydrochemical Differences Exhibit Distinct Fungal Communities

**DOI:** 10.3389/fmicb.2016.00666

**Published:** 2016-05-09

**Authors:** Ali Nawaz, Witoon Purahong, Robert Lehmann, Martina Herrmann, Kirsten Küsel, Kai U. Totsche, François Buscot, Tesfaye Wubet

**Affiliations:** ^1^Helmholtz Centre for Environmental Research – UFZ, Department of Soil EcologyHalle (Saale), Germany; ^2^Department of Biology, University of LeipzigLeipzig, Germany; ^3^Institute of Geosciences, Friedrich Schiller University JenaJena, Germany; ^4^Institute of Ecology, Friedrich Schiller University JenaJena, Germany; ^5^German Centre for Integrative Biodiversity Research (iDiv) Halle-Jena-LeipzigLeipzig, Germany

**Keywords:** fungal communities, ITS gene, pyrosequencing, karst, Upper Muschelkalk groundwater, Hainich CZE

## Abstract

Fungi are one important group of eukaryotic microorganisms in a diverse range of ecosystems, but their diversity in groundwater ecosystems is largely unknown. We used DNA-based pyro-tag sequencing of the fungal internal transcribed spacer (ITS) rDNA gene to investigate the presence and community structure of fungi at different sampling sites of two superimposed limestone aquifers ranging from 8.5 to 84 m depth in the newly established Hainich Critical Zone Exploratory (Hainich CZE). We detected a diversity of fungal OTUs in groundwater samples of all sampling sites. The relative percentage abundance of Basidiomycota was higher in the upper aquifer assemblage, whilst Ascomycota dominated the lower one. In parallel to differences in the hydrochemistry we found distinct fungal communities at all sampling sites. Classification into functional groups revealed an overwhelming majority of saprotrophs. Finding taxa common to all analyzed groundwater sites, point to a groundwater specific fungal microbiome. The presence of different functional groups and, in particular plant and cattle pathogens that are not typical of subsurface habitats, suggests links between the surface and subsurface biogeosphere due to rapid transportation across the fracture networks typical of karstic regions during recharge episodes. However, further studies including sampling series extended in both time and space are necessary to confirm this hypothesis.

## Introduction

Fungi are one of the most important ecological groups of eukaryotic microorganisms and with 1.5–1.6 million species (Hawksworth, 1991, 2001) they contribute significantly to microbial diversity on earth (O’Brien et al., 2005; Hibbett et al., 2007). The diversity estimates of fungi have been primarily based on cultures isolated from soils and plants in selected terrestrial habitats, while strains from aquatic ecosystems such as freshwater and marine habitats and their sediments have been less accounted (Richards et al., 2012). Knowledge about the complexity of fungal diversity at higher taxonomic levels is increasing with the advent of powerful molecular techniques to analyze environmental DNA. By sequencing internal transcribed spacer (ITS) genes of fungal rDNA O’Brien et al. (2005) have suggested revising the global fungal diversity estimate of Hawksworth up to 3.5–5.1 million.

In terrestrial ecosystems, fungi are a key component of microbial communities and play vital ecological roles as decomposers of dead organic matter, mutualists, or pathogens of plants and animals. Likewise in aquatic ecosystems, their roles in attenuation of contamination (Yagi et al., 2010), decomposition of dead organic matter to recycle nutrients and their potential for interacting with other organisms either as symbionts or pathogens make them one of the key drivers of aquatic food web dynamics (Wurzbacher et al., 2010).

From an ecological point of view, karstified carbonate-rock aquifers are open systems that import matter, energy, and various microorganisms during recharge events from the surface (Akob and Kusel, 2011). As a consequence, they constitute heterogeneous habitats that support a wide range of life forms (Goldscheider et al., 2006). Furthermore, they constitute a major source of water for domestic, agricultural, and industrial purposes (Lin et al., 2012). Specifically, karstic landscapes cover 20–25% of the earth’s ice-free land (Ford and Williams, 2013) and provide drinking water for almost 1.6 billion people worldwide (Martin and White, 2008). Karstic regions are characterized by their high porosity and permeability due to widened factures and cavities that favor accelerated transport from the surface to the subsurface (Anaya et al., 2014). Therefore, karstified limestone aquifers represent a complex system with sub-compartments that can host specific microbial communities and also offer a model system that is convenient to detect microorganism transport during recharge events.

There are numerous studies of fungal diversity in terrestrial (Tedersoo et al., 2014) as well as in aquatic ecosystems like the deep sea (Le Calvez et al., 2009; Nagano and Nagahama, 2012; Singh et al., 2012a,b; Xu et al., 2014), freshwater streams (Zeglin, 2015), and lakes (Wurzbacher et al., 2010). But for groundwater limestone aquifers, high throughput sequencing-based analyses of diversity, community composition and their ecological roles are scarce. Apart from fungal detection within 18S based eukaryote clone libraries from groundwater (Risse-Buhl et al., 2013), only direct microscopic observations of spores (Krauss et al., 2003) or from cultures (Lategan et al., 2012) in contaminated or managed aquifers are actually available.

In this study, we aimed to fill the knowledge gap about fungi in karstic aquifers by using culture-independent techniques. We used DNA-based high-throughput pyro-tag sequencing analysis of the fungal ITS rDNA gene to characterize fungi and their functional groups in groundwater samples from two superimposed limestone aquifer assemblages in the newly established Hainich Critical Zone Exploratory (Hainich CZE). These aquifers display marked differences with respect to their oxygen and nitrate contents. We hypothesized that (i) fungal community structures differ between the accessible domains of the two aquifer assemblages, (ii) but also share common taxa in the context of the karst landscape. Additionally, characterizing the functional groups of the detected taxa might provide valuable insights of a possible transport of fungi through the karst system.

## Materials and Methods

### Study Site and Groundwater Sampling

**FIGURE 1:**
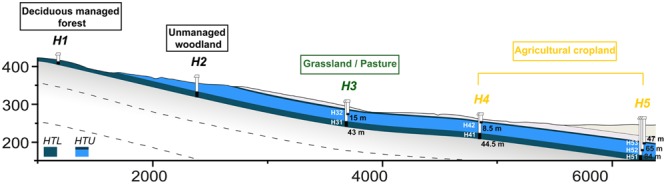
**Graphical illustration of groundwater wells in the Hainich Critical Zone Exploratory (Hainich CZE) at site H3 (located in grassland/pasture), H4 and H5 (located in agriculture/cropland) with their distances from the surface.** The illustration is 4X vertically exaggerated. Groundwater samples were collected and analyzed from the sampling sites; H31, H41, and H51 (in Hainich transect lower – HTL aquifer assemblage) and H32, H43, H52, and H53 (in Hainich transect upper – HTU aquifers assemblage). Adapted base figure from Küsel et al. (2016).

The study was conducted in the newly established Hainich CZE located in the Hainich region in the northwest of the federal state of Thuringia, Germany described in Küsel et al. (2016). Briefly, with a total area of ∼16,000 hectares (with 13,000 hectares of forest), Hainich National Park is the largest connected deciduous forest in German and the Hainich CZE follows the eastern slope of the Hainich range with a mean inclination of approximately 2°. The geological units that build soils bedrocks and aquifer/aquitard alternations of the CZE area belongs lithostratigraphically to the German Triassic subgroups Middle and Upper Muschelkalk (mainly marlstones, limestones) in the east and Lower Keuper (clay/siltstones) in the western part. A groundwater monitoring well transect (51°6.20′N 10°23.82′E–51°7.17′N 10°28.16′E) was constructed as from summer 2010 as a central feature of the Hainich CZE. This monitoring well transect, oriented in a W–E direction, following the presumed discharge direction, is ∼6 km long and covers the land use types forest (H1, H2), grassland/pasture (H3) and agricultural cropland (H4 and H5; **Figure 1**).

Groundwater samples were collected during a regular 4-weekly sampling campaign from seven permanently water-bearing monitoring wells namely H32, H43, H52, and H53 (Hainich transect upper aquifer assemblage – HTU, Meissner formation, Upper Muschelkalk) and H31, H41, H51 (Hainich transect lower aquifer assemblage – HTL, Trochitenkalk formation, Upper Muschelkalk). Recently, Opitz et al. (2014) and Herrmann et al. (2015) reported that the HTU aquifers are oxygen-deficient with low nitrate concentrations, whereas the HTL aquifer are oxygen-rich with higher nitrate concentrations. Before sampling, the groundwater extraction, using a submersible sampling pump (MP1, Grundfos, Denmark), was continued until a specific volume had been extracted and the physiochemical parameters had stabilized. For nucleic acid extractions and other subsequent molecular analysis, groundwater was transferred into sterile glass bottles and transported to the laboratory at 4°C. Approximately 5–6 l of groundwater were filtered through 0.2 μm, PES filters (Supor, Pall Corporation, Port Washington, NY, USA), which took about 40–90 min per filter. Groundwater samples were kept cold during the filtration process. The filters were then transferred into sterile reaction tubes, frozen on dry ice within 1 min and stored at -80°C until nucleic acid extraction was conducted.

### Physiochemical Analysis

For all water samples, we measured temperature, pH, dissolved oxygen concentration (DO) and specific electrical conductivity (EC) using a flow-through cell, digital sensors and multi parameter meter (Multi-3430 IDS, WTW, Weilheim, Germany). Concentration of sulfate ions was determined by ion chromatography (IC 20 system, Dionex, Sunnyvale, CA, USA) equipped with an IonPac AS11-HC column and an IonPac AG11-HC precolumn, and total organic carbon (TOC) concentrations were determined using a TOC analyzer (Analytik Jena, Jena, Germany).

### DNA Extraction, Amplicon Library, and Sequencing

Extraction of the genomic DNA was carried out from the filter disks (one filter for each of the seven sites) following the method described in Church et al. (2010). The fungal ITS rDNA region was amplified using the primer combination ITS1F (Gardes and Bruns, 1993) and ITS4 (White et al., 1990) to generate amplicon libraries. PCR products were checked by gel electrophoresis and purified using a NucleoSpin extract II kit (Macherey-Nagel, Germany) according to the manufacturer’s protocol. Barcodes and linker sequences were added in a second PCR performed by GATC-biotech (Konstanz, Germany). Amplicons were sequenced by 454-pyrosequencing on a Roche GS FLX genome sequencer system by GATC-biotech, following the manufacturer’s protocol.

### Sequence Processing

Sequential bioinformatic analysis was performed to filter out high quality reads of the sequences generated by pyrosequencing. The raw reads were first demultiplexed and quality trimmed using the MOTHUR software (v 1.33.3; Schloss et al., 2009). Briefly, reads were binned and quality filtered based on the sample barcodes with a maximum of one mismatch, the forward primer with a maximum of four mismatches, a minimum length of 400 nt, a minimum average quality score of 30 Phred, containing homopolymers with a maximum length of 8 nt, and without any ambiguous nucleotides. Chimeras were removed using UCHIME (Edgar et al., 2011) as implemented in MOTHUR. The sequence reads were normalized to the lowest number of reads per sample. Unique sequences were sorted by decreasing abundances and were clustered into operational taxonomic units (OTUs) using the CD-HIT-EST (Fu et al., 2012) at a threshold of 97% sequence similarity. Fungal ITS OTU representative sequences were first classified against the dynamic version of the UNITE database (Koljalg et al., 2013). Non-target OTUs were removed from the dataset and the fungal sequences were further classified against the full version of the UNITE database in order to improve their taxonomic annotation. In order to assess the effect of rare taxa (singletons, doubletons, and tripletons), which potentially might originate from artificial sequences (Kunin et al., 2010), we performed a Mantel test using Bray–Curtis dissimilarities to assess the correlations between the whole matrix and a matrix excluding the rare OTUs. The result indicated that the removal of rare OTUs from the total community had no effect on the fungal community composition (*R* = 0.9948, *P* = 0.001). Thus we removed the rare taxa and used the dominant taxa matrix for further statistical analysis. Finally, representative sequences of the dominant fungal OTUs were assigned into functional or ecological groups on the basis of sequence similarity using the default parameters of the GAST algorithm (Huse et al., 2008) to evaluate them against the reference dataset provided by Tedersoo et al. (2014). Total numbers of reads in the different steps of the bioinformatic workflow are presented in the **Table 2**.

### Potential Transport of Fungi

We used the number of fungal OTUs present in the first sampling site and the number of those fungal OTUs in last sampling site as an estimation of potential horizontal and vertical transport of fungi within and between the aquifers. For horizontal transport, sites H32 and H53 in the upper aquifer and sites H31 and H51 in the lower aquifer were used as the first and last sites. Whereas, for vertical transport, sites H31 and H32, H43 and H41, H53 and H51 were taken as first and last sites for sites H3, H4, and H5, respectively. Additionally, we also used the non-surface lifestyles of the detected fungal OTUs in the aquifers as an indictor of the potential transport.

### Nucleotide Accession Number

The Fungal ITS rDNA pyrosequencing data are deposited in the European Nucleotide Archive (ENA) under the study number PRJEB12647.

### Statistical Analysis

The R software-3.2.0 (R Development Core Team, 2015) and PAST software-v.2.17 (Hammer et al., 2001) were used for the data analysis. All the statistical analysis was carried out using the relative abundance values of OTUs. Sample based rarefaction curves (Hurlbert, 1971) were generated for all the samples from the upper and lower aquifers to account for sampling effort by using the function rarefy in the vegan package (Oksanen et al., 2015) implemented in R. Similarity percentages (SIMPER) analysis based on Bray–Curtis dissimilarity measures was used to calculate the pairwise and overall dissimilarity between all the sites in upper and lower aquifers and the top 10 fungal OTUs that contributed the most to the observed overall dissimilarity using PAST. UPGMA (Unweighted Pair Group Method with Arithmetic Mean) clustering based on the Bray–Curtis similarity index was calculated to determine possible clustering of the different sites from the two aquifers. Fungal communities from the upper and lower aquifers were graphed in a two-dimensional NMDS (Non-metric multidimensional scaling) ordination based on Bray–Curtis dissimilarity matrices using PAST.

## Results

### Hydrogeology and Hydrochemistry of the Aquifers

The physiochemical parameters of the groundwater samples are summarized in **Table 1**. We found that water samples from the two aquifer assemblages have neutral pH ranging from 7.1 to 7.3. Oxygen concentration in the groundwater wells of the upper aquifers ranged from 3.2 mg L^-1^ in the uphill location (H3) to 0.0 mg L^-1^ in the anoxic domains in the presumed discharge direction. The lower, oxic aquifer contained up to 8.4 mg L^-1^ (H3) dissolved oxygen. The highest concentrations of sulfate (1.39 mmol L^-1^) were observed at site H5 in the lower aquifer. Ion contents (measured as EC), TOC, and DOC concentrations did not differ markedly, except the sulfate-caused elevated EC in H51.

**Table 1:** Physiochemical parameters of groundwater samples from the upper and lower aquifers; sampling campaign September 2013.

Site	Aquifer top depth (m)	pH	DO (mg L^-1^)	DOC (μmol L^-1^)	TOC (μmol L^-1^)	SO_4_^2-^ concentration (mmol L^-1^)	EC (T_ref_ 25°C) (μS cm^-1^)
Upper aquifer
H32	15	7.3	3.2	2.54	2.72	0.5	760
H43	8.5	7.1	0	2.89	3.28	0.2	ND
H52	65	7.2	0	1.72	1.77	0.5	780
H53	47	7.3	0	1.42	1.81	0.4	725
Lower aquifer
H31	43	7.2	8.4	2.89	3.32	0.54	740
H41	44.5	7.2	4.6	2.07	2.50	0.57	ND
H51	84	7.1	2.4	2.24	2.50	1.39	1049

### Overview of Fungal ITS Dataset

A total of 149,769 raw reads were generated by 454-pyrosequencing from the seven samples collected from the upper and lower aquifers at sites H3, H4, and H5. As shown in **Table 2**, the trimming and the removal of potential chimeric sequences resulted in a final total of 113,798 reads, ranging from 11,248 to 20,890 reads per sample. Therefore, the number of reads was normalized to 11,248 reads per sample. Further sequence clustering at the ≥97% sequence similarity level followed by non-target sequence removal resulted in separating out a total of 84,939 OTUs. Sequences representing the rare taxa (single-, double-, and triple-tons), a total of 1,448, were removed from the dataset.

**Table 2:** Total number of reads at the different stages of the bioinformatic workflow.

Sample	Raw	Trimmed	Chimeras removed	Sub-sampled	Rare OTUs removed	Fungal OTUs only
H31	18926	14805	14078	11248	11211	11139
H32	15735	13759	13202	11248	9215	9113
H41	18232	13025	11248	11248	10546	10005
H43	16884	14901	13522	11248	11248	11162
H51	19267	15556	13598	11248	10921	10724
H52	18562	15031	13332	11248	11074	10795
H53	23860	21673	20890	11248	9480	9349
**Minimum**	15735	13025	11248	11248	9215	9113
**Average**	18721.1	15390.8	14224.8	11248.0	10617.4	10436.4
**Maximum**	23860	21673	20890	11248	11248	11204
**Total**	149769	123126	113798	89984	84939	83491

### Distribution Patterns of Fungi in the Upper and Lower Aquifers

**FIGURE 2:**
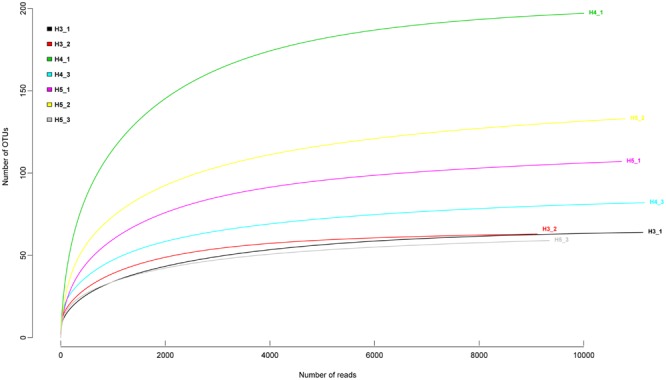
**Rarefaction curves for fungal amplicon libraries from all the sites in the upper and lower aquifer assemblages describing the number of fungal OTUs defined at the ≥97% sequence similarity as a function of the number of reads**.

**FIGURE 3:**
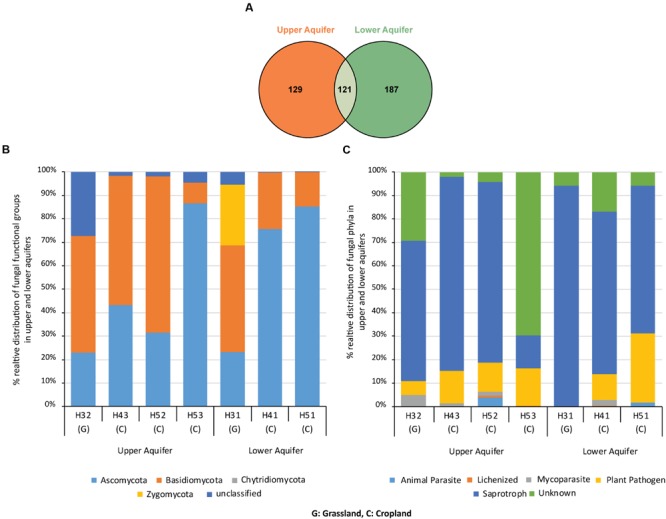
**Venn diagram **(A)** illustrating the number of fungal OTUs exclusively found in the upper and lower aquifer assemblages and OTUs that were found in both aquifers.** Relative distribution of fungal phyla **(B)** and different fungal functional groups **(C)** found in upper and lower aquifer assemblages in the Hainich CZE.

Sample-based rarefaction curves from all samples of the two aquifers reached saturation (**Figure 2**). Aquifer-specific and shared fungal OTUs were represented in a Venn diagram (**Figure 3A**). We found 129 and 187 OTUs exclusively present in the upper and lower aquifer, respectively, whilst 121 fungal OTUs were shared between these two aquifer assemblages.

Classification of the fungal OTU’s at phylum level showed the presence of three dominant fungal phyla in the dataset, i.e., Ascomycota, Basidiomycota, and Zygomycota (**Figure 3B**). Basidiomycota dominated the upper aquifer with 50, 55, and 67% relative abundance at sites H32, H43, and H52, respectively. Tremellomycetes (Basidiomycota) was the dominant fungal class in the upper aquifers. In contrast, at site H53 in the upper aquifer the phylum Ascomycota was dominant with 87% relative abundance (Basidiomycota accounted for only 9%).

In the lower aquifer, Ascomycota at sites H51 and H41 had the highest relative abundances of 85 and 76%, respectively, with Sordariomycetes, and Leotiomycetes as the dominant classes. Interestingly, site H31 in the lower aquifer exhibited a unique pattern with more even distribution of the proportions of the three fungal phyla (Ascomycota, 23%; Basidiomycota, 46%; Zygomycota, 26%). In addition, the highest numbers of unclassified OTUs were present in H32 of the upper aquifer assemblage.

### Fungal Community Structures in the Upper and Lower Aquifers

The overall dissimilarity between the fungal communities of the upper and lower aquifers calculated by SIMPER analysis was high (81.36%). The top 10 OTUs significantly contributed to the observed dissimilarity between the fungal communities of the upper and lower aquifers and could be considered to be drivers of fungal community structural change in these two aquifers are summarized in **Table 3**. The OTUs that contributed the most (16% of the total dissimilarity) were OTU_003 and OTU_005 identified as Ascomycota and Basidiomycota species, respectively, with the highest relative abundance (14 and 10%) in the upper aquifer. While in the lower aquifer, OTU_009 (Sordariomycetes) and OTU_010 (*Mortierellaceae*), belonging to the phyla Ascomycota and Zygomycota, were more abundant (10 and 9%). Together, these two OTUs – 009 and 010 accounted for 11.4% of the overall dissimilarity.

**Table 3:** Top 10 fungal OTUs that contribute the most to the observed difference in the fungal communities of the upper and lower aquifer assemblages based on SIMPER analysis.

Fungal OTU	Identified fungal taxa	Average dissimilarity	Contribution %	Mean abundance upper aquifer	Mean abundance lower aquifer
Otu_0003	Ascomycota	7.921	9.736	14%	4%
Otu_0005	Tremellales (B)	5.166	6.349	10%	1%
Otu_0009	Sordariomycetes (A)	4.938	6.069	0%	10%
Otu_0010	*Mortierellaceae* (Z)	4.32	5.309	0%	9%
Otu_0002	Malasseziales (B)	4.204	5.167	7%	5%
Otu_0004	Unclassified Fungus	4.063	4.994	8%	2%
Otu_0006	*Cryptococcus victoria* (B)	4.005	4.923	2%	9%
Otu_0011	*Monilinia fructigena* (A)	3.128	3.845	0%	6%
Otu_0001	*Davidiella* (A)	2.712	3.333	10%	12%
Otu_0007	*Malassezia restricta* (B)	2.234	2.746	2%	5%
Otu_0012	*Stereum* (B)	2.089	2.568	4%	0%

*A, Ascomycota; B, Basidiomycota; Z, Zygomycota; Un, unclassified.*

**FIGURE 4:**
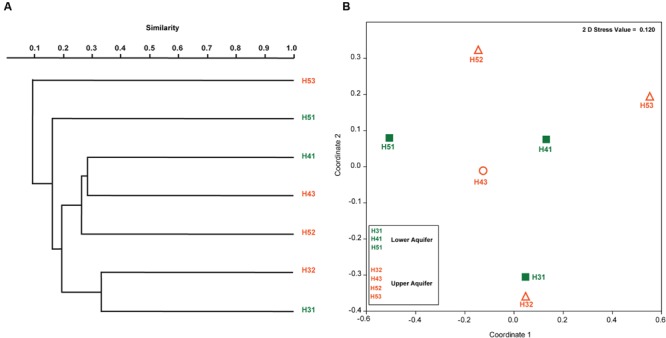
**Paired grouped (UPGMA) clustering of fungal OTUs found in the upper and lower aquifer assemblages in the Hainich CZE based on Bray–Curtis similarity index **(A)** and two-dimensional NMDS ordination of fungal community structures from upper and lower aquifers (B)**.

Paired group (UPGMA) clustering analysis using Bray–Curtis similarity indices was performed to identify possible clustering of sampling sites in the upper and lower aquifers on the basis of similarity with respect to fungal community structure. The results indicated that there was no distinct clustering pattern of the sampling sites from the two aquifer assemblages (**Figure 4A**). Instead, it revealed that none of the sites formed any true cluster with high similarity values regardless of their aquifer (upper and lower) or site (H3, H4, and H5). A two-dimensional non-metric multidimensional scaling (NMDS) ordination (**Figure 4B**) based on the Bray–Curtis distance matrix indicated that sites H31 and H32 were in close proximity but there was only 30% similarity between them (**Figure 4A**). The pairwise average dissimilarities between all the samples are given in **Table 4**. The individual and cumulative contributions of different fungal OTUs in all the pairwise dissimilarities calculated with SIMPER based on the Bray–Curtis similarity index, along with their taxonomic affiliation are given in Supplementary Table S1.

**Table 4:** Pairwise average dissimilarities between all the samples from the upper and lower aquifers based on Bray–Curtis similarity matrix.

	H31	H32	H41	H43	H51	H52	H53
H31	–						
H32	66.83	–					
H41	73.82	84.18	–				
H43	74.23	76.95	71.68	–			
H51	85.76	86.12	80.85	83.57	–		
H52	85.44	89.08	74.97	72.50	83.20	–	
H53	92.38	92.28	79.53	92.93	94.21	92.09	–

Furthermore, it can also be inferred from the data presented in Supplementary Table S1 that at each sampling site different fungal OTUs dominated the upper and lower aquifer. For instance, at site H3 (grassland/pasture), fungal OTUs belonging to Russulales sp. were dominant in the upper aquifer, whereas OTUs belonging to family *Mortierellaceae* and genera *Cryptococcus* and *Malassezia* were frequently detected in the lower aquifer. At site H4 (agricultural cropland), the most commonly detected fungal genera in the upper aquifer were *Stereum, Mycena, Xanthoria*, and *Davidiella*. In contrast, the lower aquifer was dominated by the genera *Blumeria, Cryptococcus*, and *Penicillium*. Similarly, at site H5 (agricultural cropland), the orders Tremellales and Filobasidales (*Cryptococcus* sp.) prevailed in the upper aquifer and *Monilinia fructigena* and *Bullera globospora* dominated in the lower aquifer.

### Functional Assignment of Fungal OTUs

Functional assignment of fungal OTUs revealed the presence of four dominant functional groups, i.e., saprotrophs (71%), plant pathogens (17%), animal parasites (1%) and lichenized fungi (1%; **Figure 3C**). Saprotrophs represented the dominant functional group at almost all the sampling sites in the two aquifer assemblages (60, 83, 77, 94, 69, and 63 at sites H32, H43, H52, H31, H41, and H51, respectively). Interestingly, at site H53 the proportion of plant pathogens (16%) and saprotrophs (14%) were almost equal and there was also a high proportion of unknowns at this site (70%).

### Potential Horizontal and Vertical Transport of Fungal OTUs

**FIGURE 5:**
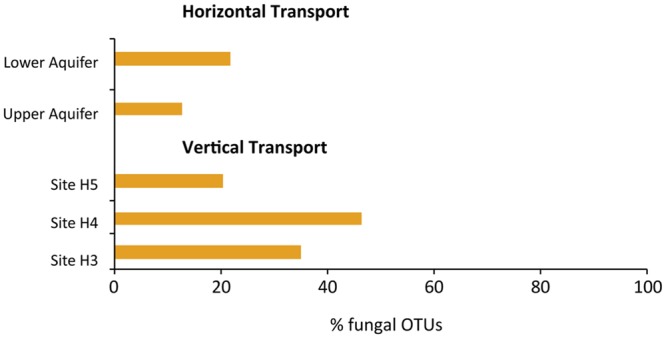
**Potential horizontal and vertical transport of fungi within and between the aquifers, estimated on the basis of number of fungal OTUs present in the first sampling site and the number of those fungal OTUs in last sampling site.** For horizontal transport, sites H32 and H53 in the upper aquifer and sites H31 and H51 in the lower aquifer were used as the first and last sites. Whereas, for vertical transport, sites H31 and H32, H43, and H41, H53, and H51 were taken as first and last sites for sites H3, H4, and H5, respectively.

Potential vertical (from top to bottom – **Figure 1** – at sites H3, H4, and H5) and horizontal (from left to right – **Figure 1** – in the upper and lower aquifers) transport of fungi was explored on the basis of shared fungal OTUs at the sampling points (**Figure 5**). The maximum potential vertical transport, i.e., 46% observed at site H4, whereas the lower aquifer had the highest potential horizontal transport of fungi, i.e., 22%. We also found fungal OTUs in upper and lower aquifers that are not specific to the subsurface habitats, i.e. wood decaying fungi (*Stereum* sp.), lichenized fungi (*Xanthoria* sp.) and wheat pathogens (*Oculimacula yallundae* and *Blumeria graminis*).

## Discussion

To our knowledge, here we present the first study with a particular focus on fungal communities in uncontaminated karstified limestone aquifers using a next generation sequencing approach, which revealed five major results. First, fungi persist in the limestone aquifers to a minimum depth of 84 m from the surface. Second, Ascomycota and Basidiomycota are the dominant fungal phyla at all the sites of the upper and lower aquifers investigated. Third, the majority of the fungi in the aquifers examined are saprotrophs. Fourth, distinct fungal communities were detected in the upper and lower aquifers and across different sampling sites along them, suggesting that the local environmental settings are a driver of the fungal community structure. Fifth, there was an indication of possible horizontal and vertical transport of fungal OTUs within and between the aquifers.

### Distribution and Functional Groups of Fungi Detected in Aquifers

In this study, we report 437 dominant fungal OTUs, which indicates that the karstic limestone aquifers in the alternating limestone and marlstone sequence of the Hainich CZE host taxonomically diverse fungal species. In the absence of further molecular data on fungal communities in other carbonate-rock aquifers, only indirect comparisons can be made with studies in related environmental settings, i.e., karst cave systems, fresh water lakes or deep groundwater fracture zones.

In the upper aquifer, the most abundant fungal class was Tremellomycetes, a nutritionally heterogeneous fungal group comprising saprotrophs, animal parasites and fungicolous species (Millanes et al., 2011). The majority of Tremellomycetes spp. are dimorphic – during their life cycle they have both a haploid unicellular yeast phase and a diploid filamentous phase (Boekhout et al., 2011). It has been reported that some of these dimorphic fungi are able to oxidize sulfur and sulfur compounds to release sulfate (Sterflinger, 2000; Reitner et al., 2006). In subsurface aquatic systems, they could participate in sulfur cycling and provide sulfate to the sulfate-reducing bacteria (Sohlberg et al., 2015).

In the lower aquifer, fungal OTUs belonging to Sordariomycetes and Leotiomycetes were dominant. Sordariomycetes, one of the largest classes in the Ascomycota (Kirk et al., 2008), are cosmopolitan fungi that can function as pathogens, endophytes of plants and mammals, mycoparasites, and saprotrophs (Zhang et al., 2006). They have not been much studied in freshwater ecosystems, but generally they are known for degrading wood in waterlogged systems (Luo et al., 2004), and their superior dispersal/attachment strategies give them a competitive advantage over other freshwater fungi (Vijaykrishna et al., 2006). The Leotiomycetes are a large group of non-lichen-forming fungi that are able to survive in various ecosystems (Wang et al., 2006). These fungi have been described as plant pathogens, fungal parasites, and terrestrial and aquatic saprobes (Wang et al., 2002), contributing to decomposition and nutrient cycling.

Interestingly, the genus *Cryptococcus* was widely distributed in both upper and lower aquifers in the Hainich CZE. The *Cryptococcus* species can grow anaerobically (Visser et al., 1990; Ekendahl et al., 2003) and have been reported previously in anoxic aquifers (Brad et al., 2008). Therefore, the presence of *Cryptococcus* species in the HTU and HTL aquifer assemblages with different amounts of dissolved oxygen provides an insight into the physiological plasticity of different fungal groups found in this study.

Although our classification of the detected fungi enabled us to detect a diversity of functional groups such as parasites or lichenized fungi, the majority of the OTUs found correspond to saprotrophs. This was somewhat expected, as in deep aquifers fungi play crucial ecological roles in the decomposition of organic substrates and the release of nutrients to other co-existing microbial communities (Sohlberg et al., 2015).

### Fungal Community Structures in Aquifers

In the Hainich CZE, the HTU aquifers were characterized in previous studies as oxygen-deficient with low nitrate concentrations, whereas the HTL aquifer was oxygen-rich with higher nitrate concentrations (Opitz et al., 2014; Herrmann et al., 2015). Küsel et al. (2016) analyzed 4 years of hydrogeochemistry data from the superimposed Hainich transect aquifer assemblages and reported higher dissolved oxygen, nitrate and redox potential in the lower than in the upper aquifer. Such variations in biogeochemical characteristics of surface and subsurface environments are known to determine variations in the community structure of microbes (Edgcomb et al., 2011; Lee et al., 2012; Soininen, 2012; Van Horn et al., 2013; Redou et al., 2015). Indeed, we found the fungal community structures in the upper and lower aquifers to be distinct across all sampling sites (**Figure 3B**). The top depths of the aquifers at our sampling sites below the surface ranged between 8.5 and 84 m and we found that distance from the surface or depth was an important factor influencing the fungal community structure. This observation is in accordance with previous studies that reported changes in the fungal community structures at different depths in different aquatic environments, i.e., sub-sea floor (Ciobanu et al., 2014; Redou et al., 2014) and deep bedrock groundwater fracture zones (Sohlberg et al., 2015).

Though in a smaller fraction, our finding of plant pathogens, lichenized fungi and animal parasites reflects the diverse lifestyles of fungi in the aquatic habitats. In the absence of any sunlight, we do not expect plants in these aquifers and the presence of plant pathogens in our dataset rather suggests that they might have been transported from the surface where their respective hosts grow. Future studies are needed, however, to investigate the presence of active plant pathogens and other plant-associated functional groups using RNA-based fungal community analysis.

### Potential Fungal Horizontal and Vertical Transport

Karstic aquifers are recharged by water from the surface after rainfall and snowmelt events (Ferrill et al., 2004; Somaratne, 2014). These recharge events not only transport water but also DOM and microorganisms (e.g., bacteria, archaea, and fungi) from the surface to the aquifers. The type of the DOM and transported microbial communities depends on the soil and vegetation cover, land use and management types, and the size of the recharge area (Lehman, 2007). Therefore, the observed differences in the fungal community structure of upper and lower aquifers along the catena of this study could partially reflect the fact that the recharge areas of two aquifers in the Hainich CZE are located at sites with different land uses and different soil characteristics (Küsel et al., 2016).

The high permeability, due to well-developed secondary porosity (including conduits) make karstified limestone aquifers an ideal system to study the processes of microbial transport from the surface to the subsurface (Anaya et al., 2014). Mathematical modeling of flow rates of water and other input signals in karst terrain has been proposed in several studies (Martin and Dean, 2001; White, 2002; Scanlon et al., 2003; Renken et al., 2008) and such flows provide a transport link between surface and subsurface (Küsel et al., 2016). Hence it is very possible that along with water, fungi are also transported through all the compartments of the CZ to the aquifers. Our finding of some fungal OTUs common to all sampling sites in aquifers, despite the physico-chemical conditions being markedly different, allows us to hypothesize that there is active transport of fungi in the superimposed aquifers that we studied. In line with this hypothesis, the variation in the percentages of commonly detected fungal OTUs at different sites along the groundwater transect is consistent with the fact that porosity and flow structures are not consistent throughout karst landscapes (Bailly-Comte et al., 2010). Nevertheless the presence and absence of a particular fungal OTU at any given site is under the influence of more than one process, i.e., transport, selection, and ecological drift. Therefore we also used the presence of fungal OTUs with non-subsurface lifestyles (i.e., plant and animal pathogens) as an indicator of the potential transport from the surface to the aquifers. For instance, at site H4, we detected *Stereum* sp. (wood decaying fungi) and *Xanthoria* sp. (lichenized fungi), forest-associated fungal taxa, that might have been transported vertically from the forest soil (H1) to the aquifer and then horizontally to sampling site H4. Similarly the presence of wheat pathogens, i.e., *Oculimacula yallundae* (Esvelt Klos et al., 2014) and *Blumeria graminis* (Belanger et al., 2003) in the upper aquifer at site H4 (agricultural cropland) also indicates possible vertical transport. To confirm this hypothesis, further spatial and especially time series need to be analyzed and, rather than DNA, RNA should be targeted in order to rule out the detection of inactive fungi or even DNA fragments. Apart from the aquifers themselves, such analyses need to consider all compartments within the critical zone, i.e., soil, subsoil, aquifers, and also to examine the consequences of major recharge (e.g., after snow melt).

## Conclusion

Our study indicates that fresh water karstic limestone aquifers host a large number of different fungal communities that have previously not been studied in unmanaged and uncontaminated aquifer systems. By combining high-resolution amplicon sequencing techniques with the infrastructure of the Hainich CZE we can contribute to the international Critical Zone Observatory platforms by filling knowledge gaps about the presence and ecological roles of fungi. Although, we cannot say much about the metabolic potential of the fungi from our DNA-based study, we suggest further RNA-based fungal community and meta-transcriptomic analysis to disentangle the functional significance of the active aquifer mycobiome.

## Author Contributions

AN, FB, and TW planned the study. AN, RL, and MH collected the samples. AN and MH performed the lab work. RL and KT contributed the physiochemical data of water samples. AN, WP, and TW analyzed the data and provided general guidance. AN wrote the manuscript. WP, KK, FB, and TW contributed in reviewing the manuscript.

## Conflict of Interest Statement

The authors declare that the research was conducted in the absence of any commercial or financial relationships that could be construed as a potential conflict of interest.

## References

[B1] Akob D. M. Kusel K. (2011). Where microorganisms meet rocks in the Earth’s Critical Zone. *Biogeosciences* 8 3531–3543. 10.5194/bg-8-3531-2011

[B2] Anaya A. A. Padilla I. Macchiavelli R. Vesper D. J. Meeker J. D. Alshawabkeh A. N. (2014). Estimating preferential flow in karstic aquifers using statistical mixed models. *Ground Water* 52 584–596. 10.1111/gwat.12084 23802921 PMC3818453

[B3] Bailly-Comte V. Martin J. B. Jourde H. Screaton E. J. Pistre S. Langston A. (2010). Water exchange and pressure transfer between conduits and matrix and their influence on hydrodynamics of two karst aquifers with sinking streams. *J. Hydrol.* 386 55–66. 10.1016/j.jhydrol.2010.03.005

[B4] Belanger R. R. Benhamou N. Menzies J. G. (2003). Cytological evidence of an active role of silicon in wheat resistance to powdery mildew (*Blumeria graminis* f. sp tritici). *Phytopathology* 93 402–412. 10.1094/PHYTO.2003.93.4.402 18944354

[B5] Boekhout T. Fonseca Á Sampaio J. P. Bandoni R. J. Fell J. W. Kwon-Chung K. J. (2011). “Chapter 100 - discussion of teleomorphic and anamorphic basidiomycetous yeasts,” in *The Yeasts* 5th Edn (London: Elsevier) 1339–1372.

[B6] Brad T. Braster M. Van Breukelen B. M. Van Straalen N. M. Roling W. F. M. (2008). Eukaryotic diversity in an anaerobic aquifer polluted with landfill leachate. *Appl. Environ. Microbiol.* 74 3959–3968. 10.1128/AEM.02820-07 18469120 PMC2446530

[B7] Church M. J. Wai B. Karl D. M. Delong E. F. (2010). Abundances of crenarchaeal amoA genes and transcripts in the Pacific Ocean. *Environ. Microbiol.* 12 679–688. 10.1111/j.1462-2920.2009.02108.x 20002133 PMC2847202

[B8] Ciobanu M. C. Burgaud G. Dufresne A. Breuker A. Redou V. Ben Maamar S. (2014). Microorganisms persist at record depths in the subseafloor of the Canterbury Basin. *ISME J.* 8 1370–1380. 10.1038/ismej.2013.250 24430485 PMC4069392

[B9] Edgar R. C. Haas B. J. Clemente J. C. Quince C. Knight R. (2011). UCHIME improves sensitivity and speed of chimera detection. *Bioinformatics* 27 2194–2200. 10.1093/bioinformatics/btr381 21700674 PMC3150044

[B10] Edgcomb V. P. Beaudoin D. Gast R. Biddle J. F. Teske A. (2011). Marine subsurface eukaryotes: the fungal majority. *Environ. Microbiol.* 13 172–183. 10.1111/j.1462-2920.2010.02318.x 21199255

[B11] Ekendahl S. O’ Neill H. A. Thomsson E. Pedersen K. (2003). Characterisation of Yeasts isolated from deep igneous rock aquifers of the Fennoscandian Shield. *Microb. Ecol.* 46 416–428. 10.1007/s00248-003-2008-5 14502418

[B12] Esvelt Klos K. L. Wetzel H. C. Murray T. D. (2014). Resistance to *Oculimacula yallundae* and *Oculimacula acuformis* is conferred by Pch2 in wheat. *Plant Pathol.* 63 400–404. 10.1111/ppa.12107

[B13] Ferrill D. A. Sims D. W. Waiting D. J. Morris A. P. Franklin N. M. Schultz A. L. (2004). Structural framework of the Edwards Aquifer recharge zone in south-central Texas. *Geol. Soc. Am. Bull.* 116 407–418. 10.1130/B25174.1

[B14] Ford D. Williams P. D. (2013). *Karst Hydrogeology and Geomorphology.* New York, NY: John Wiley & Sons.

[B15] Fu L. M. Niu B. F. Zhu Z. W. Wu S. T. Li W. Z. (2012). CD-HIT: accelerated for clustering the next-generation sequencing data. *Bioinformatics* 28 3150–3152. 10.1093/bioinformatics/bts565 23060610 PMC3516142

[B16] Gardes M. Bruns T. D. (1993). ITS primers with enhanced specificity for basidiomycetes–application to the identification of mycorrhizae and rusts. *Mol. Ecol.* 2 113–118. 10.1111/j.1365-294X.1993.tb00005.x 8180733

[B17] Goldscheider N. Hunkeler D. Rossi P. (2006). Review: microbial biocenoses in pristine aquifers and an assessment of investigative methods. *Hydrogeol. J.* 14 926–941. 10.1007/s10040-005-0009-9

[B18] Hammer Ø Harper D. A. T. Ryan P. D. (2001). PAST: Paleontological statistics software package for education and data analysis. *Palaeontol. Electron.* 4 1–9.

[B19] Hawksworth D. L. (1991). The fungal dimension of biodiversity - magnitude, significance, and conservation. *Mycol. Res.* 95 641–655. 10.1016/S0953-7562(09)80810-1

[B20] Hawksworth D. L. (2001). The magnitude of fungal diversity: the 1.5 million species estimate revisited. *Mycol. Res.* 105 1422–1432. 10.1017/S0953756201004725

[B21] Herrmann M. Rusznyák A. Akob D. M. Schulze I. Opitz S. Totsche K. U. (2015). Large fractions of CO_2_-fixing microorganisms in pristine limestone aquifers appear to be involved in the oxidation of reduced sulfur and nitrogen compounds. *Appl. Environ. Microbiol.* 81 2384–2394. 10.1128/AEM.03269-14 25616797 PMC4357952

[B22] Hibbett D. S. Binder M. Bischoff J. F. Blackwell M. Cannon P. F. Eriksson O. E. (2007). A higher-level phylogenetic classification of the Fungi. *Mycol. Res.* 111 509–547. 10.1016/j.mycres.2007.03.004 17572334

[B23] Hurlbert S. H. (1971). The nonconcept of species diversity: a critique and alternative parameters. *Ecology* 52 577–586. 10.2307/1934145 28973811

[B24] Huse S. M. Dethlefsen L. Huber J. A. Welch D. M. Relman D. A. Sogin M. L. (2008). Exploring microbial diversity and taxonomy using SSU rRNA hypervariable tag sequencing. *PLoS Genet.* 4:e1000255 10.1371/journal.pgen.1000255 PMC2577301 19023400

[B25] Kirk P. M. Cannon P. F. David J. C. Stalpers J. A. (2008). *Ainsworth & Bisby’s Dictionary of the Fungi.* Surrey: CABI Bioscience.

[B26] Koljalg U. Nilsson R. H. Abarenkov K. Tedersoo L. Taylor A. F. S. Bahram M. (2013). Towards a unified paradigm for sequence-based identification of fungi. *Mol. Ecol.* 22 5271–5277. 10.1111/mec.12481 24112409

[B27] Krauss G. Sridhar K. R. Jung K. Wennrich R. Ehrman J. Barlocher F. (2003). Aquatic hyphomycetes in polluted groundwater habitats of central Germany. *Microb. Ecol.* 45 329–339. 12704555 10.1007/s00248-003-0001-7

[B28] Kunin V. Engelbrektson A. Ochman H. Hugenholtz P. (2010). Wrinkles in the rare biosphere: pyrosequencing errors can lead to artificial inflation of diversity estimates. *Environ. Microbiol.* 12 118–123. 10.1111/j.1462-2920.2009.02051.x 19725865

[B29] Küsel K. Totsche K. U. Trumbore S. E. Lehmann R. Steinhäuser C. Herrmann M. (2016). How deep can surface signals be traced in the critical zone? Merging biodiversity with biogeochemistry research in a central German Muschelkalk landscape. *Front. Earth Sci.* 4:32.

[B30] Lategan M. J. Torpy F. R. Newby S. Stephenson S. Hose G. C. (2012). Fungal diversity of shallow aquifers in Southeastern Australia. *Geomicrobiol. J.* 29 352–361. 10.1080/01490451.2011.559306

[B31] Le Calvez T. Burgaud G. Mahe S. Barbier G. Vandenkoornhuyse P. (2009). Fungal diversity in deep-sea hydrothermal ecosystems. *Appl. Environ. Microbiol.* 75 6415–6421. 10.1128/AEM.00653-09 19633124 PMC2765129

[B32] Lee C. K. Barbier B. A. Bottos E. M. Mcdonald I. R. Cary S. C. (2012). the inter-valley soil comparative survey: the ecology of dry valley edaphic microbial communities. *ISME J.* 6 1046–1057. 10.1038/ismej.2011.170 22170424 PMC3329106

[B33] Lehman R. M. (2007). “Microbial distributions and their potential controlling factors in terrestrial subsurface environments,” in *The Spatial Distribution of Microbes in the Environment* eds Rima F. Aaron M. (Berlin: Springer) 135–178.

[B34] Lin X. Mckinley J. Resch C. T. Kaluzny R. Lauber C. L. Fredrickson J. (2012). Spatial and temporal dynamics of the microbial community in the Hanford unconfined aquifer. *ISME J.* 6 1665–1676. 10.1038/ismej.2012.26 22456444 PMC3498919

[B35] Luo J. Yin J. F. Cai L. Zhang K. Q. Hyde K. D. (2004). Freshwater fungi in Lake Dianchi, a heavily polluted lake in Yunnan, China. *Fungal Div.* 16 93–112.

[B36] Martin J. B. Dean R. W. (2001). Exchange of water between conduits and matrix in the *Floridan aquifer*. *Chem. Geol.* 179 145–165. 10.1016/S0009-2541(01)00320-5

[B37] Martin J. B. White W. B. (eds) (2008). *Frontiers of Karst Research, Special Publication 13.* Lessburg, VA: Karst Warer Institute.

[B38] Millanes A. M. Diederich P. Ekman S. Wedin M. (2011). Phylogeny and character evolution in the jelly fungi (Tremellomycetes, Basidiomycota, Fungi). *Mol. Phylogenet. Evol.* 61 12–28. 10.1016/j.ympev.2011.05.014 21664282

[B39] Nagano Y. Nagahama T. (2012). Fungal diversity in deep-sea extreme environments. *Fungal Ecol.* 5 463–471. 10.1007/978-3-642-23342-5_9 22222832

[B40] O’Brien H. E. Parrent J. L. Jackson J. A. Moncalvo J. M. Vilgalys R. (2005). Fungal community analysis by large-scale sequencing of environmental samples. *Appl. Environ. Microbiol.* 71 5544–5550. 10.1128/AEM.71.9.5544-5550.2005 16151147 PMC1214672

[B41] Oksanen J. Blanchet F. Kindt R. Legendre P. O’ Hara R. Simpson G. (2015). *Vegan: Community Ecology Package*. *R Package Version 2.3–1.*

[B42] Opitz S. Küsel K. Spott O. Totsche K. U. Herrmann M. (2014). Oxygen availability and distance to surface environments determine community composition and abundance of ammonia-oxidizing prokaroytes in two superimposed pristine limestone aquifers in the Hainich region, Germany. *FEMS Microbiol. Ecol.* 90 39–53. 10.1111/1574-6941.12370 24953994

[B43] R Development Core Team (2015). *R: A Language and Environment for Statistical Computing.* Vienna: R Foundation for Statistical Computing.

[B44] Redou V. Ciobanu M. C. Pachiadaki M. G. Edgcomb V. Alain K. Barbier G. (2014). In-depth analyses of deep subsurface sediments using 454-pyrosequencing reveals a reservoir of buried fungal communities at record-breaking depths. *FEMS Microbiol. Ecol.* 90 908–921. 10.1111/1574-6941.12447 25348233

[B45] Redou V. Navarri M. Meslet-Cladiere L. Barbier G. Burgaud G. (2015). Species richness and adaptation of marine fungi from deep-subseafloor sediments. *Appl. Environ. Microbiol.* 81 3571–3583. 10.1128/AEM.04064-14 25769836 PMC4407237

[B46] Reitner J. Schumann G. Pedersen K. (2006). Fungi in subterranean environments. *Fungi Biogeochem. Cycles* 24:377 10.1017/CBO9780511550522.017

[B47] Renken R. A. Cunningham K. J. Shapiro A. M. Harvey R. W. Zygnerski M. R. Metge D. W. (2008). Pathogen and Chemical Transport in the Karst Limestone of the *Biscayne aquifer*: 1. Revised conceptualization of groundwater flow. *Water Resour. Res.* 44.

[B48] Richards T. A. Jones M. D. Leonard G. Bass D. (2012). Marine fungi: their ecology and molecular diversity. *Annu. Rev. Mar. Sci.* 4 495–522. 10.1146/annurev-marine-120710-100802 22457985

[B49] Risse-Buhl U. Herrmann M. Lange P. Akob D. M. Pizani N. Schonborn W. (2013). Phagotrophic protist diversity in the groundwater of a karstified aquifer - morphological and molecular analysis. *J. Eukaryot. Microbiol.* 60 467–479. 10.1111/jeu.12054 23808986

[B50] Scanlon B. R. Keese K. Reedy R. C. Simunek J. Andraski B. J. (2003). Variations in flow and transport in thick desert vadose zones in response to paleoclimatic forcing (0-90 kyr): field measurements, modeling, and uncertainties. *Water Resour. Res.* 39 1179 10.1029/2002WR001604

[B51] Schloss P. D. Westcott S. L. Ryabin T. Hall J. R. Hartmann M. Hollister E. B. (2009). Introducing mothur: open-source, platform-independent, community-supported software for describing and comparing microbial communities. *Appl. Environ. Microbiol.* 75 7537–7541. 10.1128/AEM.01541-09 19801464 PMC2786419

[B52] Singh P. Raghukumar C. Meena R. M. Verma P. Shouche Y. (2012a). Fungal diversity in deep-sea sediments revealed by culture-dependent and culture-independent approaches. *Fungal Ecol.* 5 543–553. 10.1016/j.funeco.2012.01.001

[B53] Singh P. Raghukumar C. Verma P. Shouche Y. (2012b). Assessment of fungal diversity in deep-sea sediments by multiple primer approach. *World J. Microbiol. Biotechnol.* 28 659–667. 10.1007/s11274-011-0859-3 22806861

[B54] Sohlberg E. Bomberg M. Miettinen H. Nyyssonen M. Salavirta H. Vikman M. (2015). Revealing the unexplored fungal communities in deep groundwater of crystalline bedrock fracture zones in Olkiluoto, Finland. *Front. Microbiol.* 6:573 10.3389/fmicb.2015.00573 PMC4460562 26106376

[B55] Soininen J. (2012). Macroecology of unicellular organisms - patterns and processes. *Environ. Microbiol. Rep.* 4 10–22. 10.1111/j.1758-2229.2011.00308.x 23757224

[B56] Somaratne N. (2014). Characteristics of point recharge in karst aquifers. *Water* 6 2782–2807. 10.3390/w6092782

[B57] Sterflinger K. (2000). Fungi as geologic agents. *Geomicrobiol. J.* 17 97–124. 10.1080/01490450050023791

[B58] Tedersoo L. Bahram M. Polme S. Koljalg U. Yorou N. S. Wijesundera R. (2014). Global diversity and geography of soil fungi. *Science* 346: 1078 10.1126/science.1256688 25430773

[B59] Van Horn D. J. Van Horn M. L. Barrett J. E. Gooseff M. N. Altrichter A. E. Geyer K. M. (2013). Factors controlling soil microbial biomass and bacterial diversity and community composition in a cold desert ecosystem: role of geographic scale. *PLoS ONE* 8:e66103 10.1371/journal.pone.0066103 PMC3688848 23824063

[B60] Vijaykrishna D. Jeewon R. Hyde K. D. (2006). Molecular taxonomy, origins and evolution of freshwater ascomycetes. *Fungal Div.* 23 351–390.

[B61] Visser W. Scheffers W. A. Vegte W. H. Van Dijken J. P. (1990). Oxygen requirements of yeasts. *Appl. Environ. Microbiol.* 56 3785–3792. 2082825 10.1128/aem.56.12.3785-3792.1990 PMC185068

[B62] Wang Z. Binder M. Hibbett D. S. (2002). A new species of Cudonia based on morphological and molecular data. *Mycologia* 94 641–650. 10.2307/3761715 21156537

[B63] Wang Z. Johnston P. R. Takamatsu S. Spatafora J. W. Hibbett D. S. (2006). Toward a phylogenetic classification of the leotiomycetes based on rDNA data. *Mycologia* 98 1065–1075. 10.3852/mycologia.98.6.1065 17486981

[B64] White T. Bruns T. Lee S. Taylor J. (1990). “Amplification and direct sequencing of fungal ribosomal RNA genes for phylogenetics,” in *PCR Protocols: A Guide to Methods and Applications* eds Innis M. Gelfand D. Shinsky J. White T. (Cambridge: Academic Press) 315–322.

[B65] White W. B. (2002). Karst hydrology: recent developments and open questions. *Eng. Geol.* 65 85–105. 10.1016/S0013-7952(01)00116-8

[B66] Wurzbacher C. M. Barlocher F. Grossart H. P. (2010). Fungi in lake ecosystems. *Aquat. Microb. Ecol.* 59 125–149. 10.3354/ame01385

[B67] Xu W. Pang K. L. Luo Z. H. (2014). High fungal diversity and abundance recovered in the deep-sea sediments of the Pacific Ocean. *Microb. Ecol.* 68 688–698. 10.1007/s00248-014-0448-8 25004994

[B68] Yagi J. M. Neuhauser E. F. Ripp J. A. Mauro D. M. Madsen E. L. (2010). Subsurface ecosystem resilience: long-term attenuation of subsurface contaminants supports a dynamic microbial community. *ISME J.* 4 131–143. 10.1038/ismej.2009.101 19776766

[B69] Zeglin L. H. (2015). Stream microbial diversity in response to environmental changes: review and synthesis of existing research. *Front. Microbiol.* 6:454 10.3389/fmicb.2015.00454 PMC4435045 26042102

[B70] Zhang N. Castlebury L. A. Miller A. N. Huhndorf S. M. Schoch C. L. Seifert K. A. (2006). An overview of the systematics of the Sordariomycetes based on a four-gene phylogeny. *Mycologia* 98 1076–1087. 10.3852/mycologia.98.6.1076 17486982

